# The activation of cardiac *dSir2*-related pathways mediates physical exercise resistance to heart aging in old *Drosophila*

**DOI:** 10.18632/aging.102261

**Published:** 2019-09-10

**Authors:** Deng-Tai Wen, Lan Zheng, Jin-Xiu Li, Kai Lu, Wen-Qi Hou

**Affiliations:** 1 Key Laboratory of Physical Fitness and Exercise Rehabilitation of Hunan Province, Hunan Normal University, Changsha 410012, Hunan Province, China; 2 Ludong University, Yantai 264025, Shan Dong Province, China

**Keywords:** exercise, heart aging, *dSir2*, oxidative stress, lipid accumulation

## Abstract

Cardiac aging is majorly characterized by increased diastolic dysfunction, lipid accumulation, oxidative stress, and contractility debility. The *Sir2/Sirt1* gene overexpression delays cell aging and reduces obesity and oxidative stress. Exercise improves heart function and delays heart aging. However, it remains unclear whether exercise delaying heart aging is related to cardiac *Sir2/Sirt1*-related pathways. In this study, cardiac *dSir2* overexpression or knockdown was regulated using the UAS/hand-Gal4 system in *Drosophila*. Flies underwent exercise interventions from 4 weeks to 5 weeks old. Results showed that either cardiac *dSir2* overexpression or exercise remarkably increased the cardiac period, systolic interval, diastolic interval, fractional shortening, SOD activity, dSIR2 protein, *Foxo*, *dSir2*, *Nmnat*, and *bmm* expression levels in the aging flies; they also notably reduced the cardiac triacylglycerol level, malonaldehyde level, and the diastolic dysfunction index. Either cardiac *dSir2* knockdown or aging had almost opposite effects on the heart as those of cardiac *dSir2* overexpression. Therefore, we claim that cardiac *dSir2* overexpression or knockdown delayed or promoted heart aging by reducing or increasing age-related oxidative stress, lipid accumulation, diastolic dysfunction, and contractility debility. The activation of cardiac *dSir2/Foxo/*SOD and *dSir2/Foxo/bmm* pathways may be two important molecular mechanisms through which exercise works against heart aging in *Drosophila*.

## INTRODUCTION

The aging population is increasing in a lot of countries, and age-related heart disease has become a major cause of cardiovascular mortality and morbidity in modern society [1, 2]. Cardiac aging is majorly characterized by increased diastolic dysfunction, accumulation of lipids, and oxidative stress [3–6]. Since the incidence of heart disease increases dramatically with age, it is important to understand the molecular mechanisms through which the heart becomes either more or less susceptible to stress. Sirtuin (SIRT1) and silent information regulator 2 (Sir2) proteins are classes of proteins that possess nicotinamide adenine dinucleotide (NAD^+^)-dependent deacetylase activity and ADP-ribosyltransferase activity, respectively, and they are evolutionarily conserved from bacteria to humans [7]. Sirt1 proteins participate in regulating cell aging, diabetes, obesity, and oxidative stress [8]. In mice hearts, SIRT1 overexpression relieves AngII-induced cardiomyocyte hypertrophy and apoptosis [9]. Up-regulation and activation of Sirt1 induced by phenylephrine are blocked by the inhibition or downregulation of AMPK, leading to improved cell survival under hypertrophic stress [10]. Besides, some studies examining the effects of resveratrol indicate that Sirt1 may have a beneficial role in failing hearts and that endogenous Sirt1 up-regulation is a protective mechanism in the early stage of heart failure [11, 12]. Sirt1 has also been reported to up-regulate Mn-SOD via hypoxia-inducible factor-2α and FoxO4, and several lines of evidence suggest that Sir2 plays a protective role against oxidative stress in cardiomyocytes and the heart. Cardiac-specific overexpression of Sirt1 induces an increase in the protein expression of catalase after exposure to paraquat. During cardiac I/R, overexpression of Sirt1 also upregulates Mn-SOD and Trx1, and attenuates oxidative stress [13–15]. Interestingly, lipid accumulation can evoke cardiac oxidative stress, inflammation and, eventually, cardiac dysfunction and heart failure [16]. Adipose *FOXO* expression protects against HFD-induced cardiac malfunction, and the expression of *FOXO* in myocardial cells autonomously protects the heart from the adverse effects of a high-fat-diet in *Drosophila* [17, 18]. Sirt1 has been found to deacetylate Foxo1 at K242, K245, and K262 and induce nuclear translocation, which may resist lipid accumulation in heart [19–21]. Therefore, the evidence suggests that *Sirt1* may be a key gene involved in regulating heart aging. However, it remains unknown whether the cardiac *Sir2* gene in flies can affect age-related heart diastolic dysfunction, lipid accumulation, and oxidative stress.

Exercise, as an inducible form of physiologic stress, represents a powerful tool in cardiac aging research. Exercise physiology has provided a wealth of knowledge into how age-related changes in cardiac structure and function translate to decreased exercise capacity, which is a strong determinant of heart failure prognosis [22]. Age-related lipid accumulation and myocardial fiber loss are important causes of pathologic myocardial hypertrophy, which can also induce lipid toxic injury of cardiomyocytes [3, 23]. Exercise training can speed up fat mobilization and promote lipid decomposition, which can effectively prevent the heart from excessive accumulation of lipids [24]. Besides, cardiac aging is characterized by concentric remodeling and decrements in diastolic functions, which are theorized to contribute to the increased risk of heart failure in older adults [6]. Since physical activity has been related to higher cardiac internal dimensions and improved diastolic function, age-associated cardiac remodeling might be an appropriate target for exercise therapy [25, 26]. Moreover, oxidative stress, defined as an excess production of reactive oxygen species (ROS) relative to antioxidant defense, has been shown to play an important role in the pathophysiology of cardiac remodeling and heart failure [27]. Meanwhile, treatment of diabetic rats with exercise significantly decreased the levels of MDA and increased the activity of SOD, GPx, and CAT compared with untreated diabetic rats; this indicated the protective effect of voluntary exercise against oxidative stress in the hearts of high-fat diet-induced type 2 diabetic rats [28, 29]. Finally, the expression of the pro-survival p-Akt protein decreased significantly with age and reduced cardiac performance. The IGF1R/PI3K/Akt survival pathway in the heart of young rats can indeed be increased through exercise training, and exercise training enhanced the SIRT longevity pathway instead of IGF1 survival signaling to improve cardiomyocyte survival [30]. In *Drosophila*, increasing evidence shows that endurance exercise or regular exercise also improves heart function and delay heart age-related phenotypes [31–36]. Therefore, the evidence indicates that exercise training can delay cardiac aging, but it remains unclear whether exercise training delaying heart aging is related to cardiac Sirt1-related pathways, and the effect of cardiac *Sir2* gene differential expression combined with exercise training on heart aging is still unknown.

To explore the relationship between cardiac *Sir2*, exercise, and cardiac aging, cardiac *Sir2* knockdown or overexpression was induced using the UAS/hand-Gal4 system in *Drosophila*. The flies underwent exercise training using a TreadWheel. The cardiac *Sir2* expression level was tested by qRT-PCR. The heart period, systolic period, diastolic period, diastolic dysfunction index, fractional shortening, diastolic diameter, and systolic diameter were measured by an M-mode trace. Next, the cardiac triacylglycerol (TAG) and *brummer (bmm)* gene expression were measured by ELISA and qRT-PCR. The cardiac SOD activity level, MDA level, and *Foxo* gene expression level were detected by ELISA and qRT-PCR. Based on these indicators, we tried to understand the relationship between cardiac *Sir2*, exercise, and cardiac aging.

## RESULTS

### Cardiac *dSir2* knockdown promotes heart aging in *Drosophila*

#### Cardiac dSir2 knockdown promotes age-related oxidative stress in aging Drosophila

**Figure 1:**
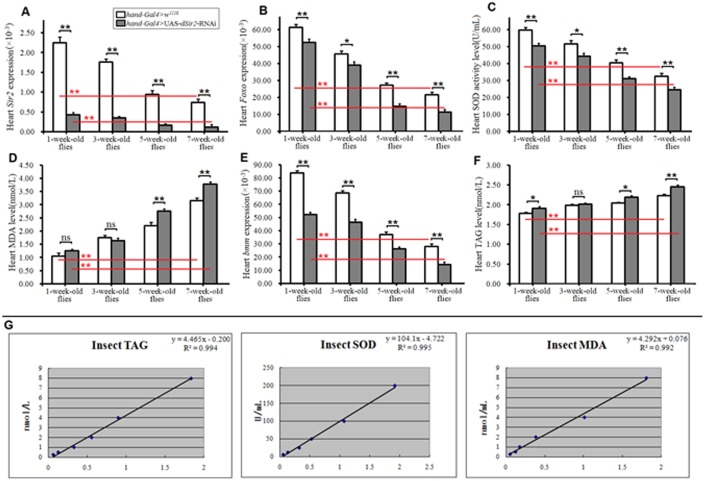
**Influence of cardiac dSir2 knockdown on heart lipid accumulation and oxidative stress.** (**A**) Cardiac dSir2 expression level. (**B**) Cardiac *Foxo* expression. (**C**) Cardiac SOD activity level. (**D**) Cardiac MDA level. (**E**) Cardiac *bmm* expression. (**F**) Cardiac TAG level. (**G**) The normal line of TAG, the normal line of SOD, and the normal line of MDA. Independent-sample *t* tests were used to assess differences between the 1-week-old and 7-week-old flies to explore the effects of aging on the heart. Independent-sample *t* tests were used to assess differences between *hand-Gal4>w^11118^* and *hand-Gal4>UAS-dSir2-RNAi* flies to explore the effects of cardiac *dSir2* knockdown on the heart. Data are represented as means ± SEM. *P<0.05; **P <0.01. Sample size was 80 hearts for each group, and measurements were taken 3 times.

The Gal4/upstream activating sequence (UAS) system is one of the most powerful tools for targeted gene expression. It is based on the properties of the yeast GAL4 transcription factor, which activates transcription of its target genes by binding to UAS *cis*-regulatory sites. In *Drosophila*, the two components are carried in separate lines, allowing for numerous combinatorial possibilities [37]. The bipartite system is commonly used in gain-of-function analysis, and by combining with the RNA interference technology, it can also be applied in loss-of-function analysis [38]. In this study, the results showed that at different ages the cardiac *dSir2* expression of *hand-Gal4>UAS-dSir2-RNAi* flies was significantly lower than that of *hand-Gal4>w^1118^* flies (P<0.01; Figure 1A). In both *hand-Gal4>w^1118^* and *hand-Gal4>UAS-dSir2-RNAi* flies, the cardiac *dSir2* expression of 7-week-old flies was lower than that of 1-week-old flies (P<0.01; Figure 1B). These results suggested that the *dSir2* gene knockdown was successfully induced by *hand-Gal4>UAS-dSir2-RNAi* in flies’ heart. Aging notably reduced heart *dSir2* expression in flies.

Superoxide dismutase (SOD) and malondialdehyde (MDA) are two common indices for evaluating the ability of eliminating oxygen free radicals [39]. A large amount of SOD exist in the body, which eliminates free radicals and has important roles in protecting the body from damage by reactive oxygen species (ROS) [40, 41] SOD catalyzes the dismutation of superoxide into oxygen and hydrogen peroxide during physiological and pathological state, including aging [42]. It has been demonstrated that the expression and activity of the SOD system are modified during aging, with reduced cell ability to counteract the oxidant molecules, and consequent weak resistance to ROS accumulation [43]. Similar to protein carbonyl production, lipids can be modified and are indicative of oxidative stress. MDA is commonly used as a marker of lipid peroxidation, and is typically accessed via the thiobarbituric acid reactive substances assay; however, this assay can be somewhat nonspecific as it can react with other aldehydes in addition to MDA. MDA is generated *in* *vivo* via peroxidation of polyunsaturated fatty acids [44]. MDA together with excessive oxyradicals attacks the cell membrane, which leads to cell necrosis [45–47]. Meanwhile, the activation of *Foxo* is associated with an increase in the expression of SOD; however, this increase is unrelated to an increase in SOD activity [48]. The *dSir2* can modulate the cellular stress response directly by deacetylating some proteins, such as Foxo, and regulating their expression [14].

In this study, we found that in flies of different ages, cardiac *dSir2* knockdown significantly reduced the SOD activity level and *Foxo* expression in the heart (P<0.05 or P<0.01; Figure 1B and 1C). It notably increased cardiac MDA level in 5-week-old and 7-week-old flies (P<0.05 or P<0.01; Figure 1D). Besides, we found that aging significantly increased the cardiac MDA level (P<0.01; Figure 1C), and it notably reduced SOD activity level and *Foxo* expression in both *hand-Gal4>w^1118^* and *hand-Gal4>UAS-dSir2-RNAi* flies (P<0.01; Figure 1B and 1E). These results suggested that cardiac *dSir2* knockdown could promote age-related oxidative damage by inhibiting *Foxo/SOD* pathway activity in aging hearts.

#### Cardiac dSir2 knockdown promotes age-related fat accumulation in aging Drosophila

With age, the heart exhibits a decrease in the number of myocytes with a concomitant increase in the size of each cardiomyocyte and an increased accumulation of lipids and areas of fibrosis [3]. The uptake of fatty acids through the sarcolemma is augmented because of an increase in the amount of transport protein and CD36, but fatty acid oxidation is decreased. Fatty acid oxidation is a major component of the energy production process as it accounts for the generation of approximately 70% of cardiac ATP [23]. Fatty acid utilization in healthy hearts is a complex process that includes several steps: FA uptake, conversion of free FA to FA-CoA, storage of FAs in triglycerides (TG), TG lipolysis, transfer of fatty acids into the mitochondria, β-oxidation, and oxidative phosphorylation for ATP production. The flawless transfer of fatty acids from cellular uptake to mitochondrial oxidation prevents the accumulation of excess lipids [49]. Our previous results suggest that a high-fat diet can accelerate the age-related downregulation of *dSir2* gene expression in *Drosophila* [35], but it remains unclear whether the cardiac *dSir2* knockdown can contribute to lipid accumulation in the heart.

In this study, cardiac TAG level and *brummer(bmm)* gene expression was measured to reflect cardiac lipid accumulation. We found that in flies 1 week, 5 weeks, and 7 weeks old, cardiac *dSir2* knockdown significantly increased the level of TAG in the heart (P<0.05 or P<0.01)(1 week old: increased by 7.3%; 5 weeks old: increased by 7.8%; and 7 weeks old: increased by 8.2%). Aging also significantly increased cardiac TAG levels in both *hand-Gal4>w^1118^* and *hand-Gal4>UAS-dSir2-RNAi* flies (P<0.01; Figure 1F). The cardiac *bmm* gene encodes the lipid storage droplet-associated TAG lipase *Brummer*, a homolog of human adipocyte triglyceride lipase (ATGL). Food deprivation or chronic *bmm* overexpression depleted organismal fat stores *in vivo*, whereas loss of *bmm* activity caused obesity in flies. Therefore, the change in *bmm* gene function is an important molecular mechanism that leads to TAG metabolic disorder [50]. In this experiment, we found that cardiac *dSir2* knockdown significantly decreased the cardiac *bmm* gene expression in flies of different ages (P<0.01) (Figure 1E). We also found that aging significantly decreased the cardiac *bmm* gene expression in both *hand-Gal4>w^1118^* and *hand-Gal4>UAS-dSir2-RNAi* flies (P<0.01) (Figure 1E). These results suggested that cardiac *dSir2* knockdown could promote age-related TAG accumulation by reducing *bmm* expression in the heart.

#### Cardiac dSir2 knockdown promotes age-related diastolic dysfunction and contractility asthenia in aging Drosophila

**Figure 2:**
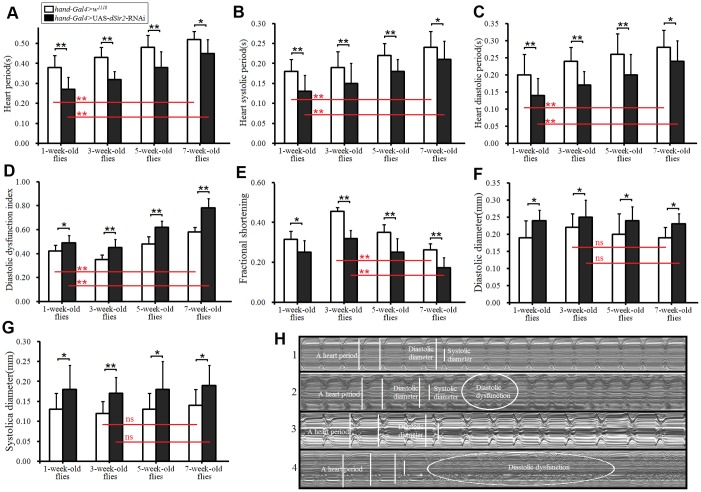
**Heart function was measured by M-mode trace.** (**A**) Heart period. (**B**) Heart systolic period. (**C**) Heart diastolic period. (**D**) Heart diastolic dysfunction index. The diastolic dysfunction index is diastolic interval standard deviation/diastolic interval median). (**E**) Fractional shortening. (**F**) Diastolic diameter. (**G**) Systolic diameter. (**H**) Microscopic image of cardiac function from M-mode trace in 5-week-old and 7-week-old *Drosophila*. 1: 5-week-old *hand-Gal4>w^11118^* flies; 2: 5-week-old *hand-Gal4>UAS-dSir2-RNAi* flies; 3: 7-week-old *hand-Gal4>w^11118^* flies; 4: 7-week-old *hand-Gal4>UAS-dSir2-RNAi* flies. It can be observed from 1, 2, 3, and 5 that the cardiac *dSir2* knockdown could reduce heart period and fractional shortening, and increase diastolic dysfunction. Independent-sample *t* tests were used to assess differences between the 1-week-old” and 7-week-old flies to explore the effects of aging on the heart. Independent-sample *t* tests were used to assess differences between the *hand-Gal4>w^11118^* and *hand-Gal4>UAS-dSir2-RNAi* flies to explore the effects of cardiac *dSir2* knockdown on the heart. Data are represented as means ± SEM. *P<0.05; **P <0.01. Sample size was 30 hearts for each group.

Age-related cardiac dysfunction is a major factor in heart failure. Diastolic dysfunction is highly prevalent, and aging is the main contributor due to impairments in active cardiac relaxation [51]. Sir2/Sirt1 is a class III histone deacetylase, and it mediates lifespan extension in model organisms and prevents apoptosis in mammalian cells. Moderate overexpression of *Sir2/Sirt1* in hearts can attenuate age-dependent increases in apoptosis/fibrosis and cardiac dysfunction [13]. However, it remains unclear whether cardiac *dSir2* knockdown can affect heart period, diastolic dysfunction, and contractility. In this study, the results showed that in both *hand-Gal4>w^1118^* and *hand-Gal4>UAS-dSir2-RNAi* flies, aging significantly increased cardiac period, systolic interval, diastolic interval, and the diastolic dysfunction index (P<0.01; Figure 2A–2D); furthermore, aging notably reduced fractional shortening (Figure 2E). Moreover, cardiac *dSir2* knockdown significantly reduced the cardiac period, systolic interval, and diastolic interval (P<0.05 or P<0.01; Figure 2A–2C), and it notably increased the diastolic dysfunction index, fractional shortening, diastolic diameter, and systolic diameter (P<0.05 or P<0.01; Figure 2D–2H). The results showed that the shortened heart period induced by *dSir2* RNAi was opposite to the increased heart period induced by aging, and this seemed to contradict the idea that dSir2 RNAi promoted heart aging. However, the effects of dSir2 RNAi and aging on heart contractility and the diastolic dysfunction index were the same. We speculate that from the perspective of the overall changes in the heart, the reduction in cardiac contractility induced by dSir2 RNAi reduced the output per stroke, and to meet the needs of the body for hemolymph, the heart shortened the heart period, increased its heart rate, and finally increased its total output. Therefore, these results suggested that cardiac *dSir2* knockdown could increase the risk of cardiac diastolic dysfunction by reducing cardiac relaxation time, which could promote the occurrence of cardiac age-related diastolic dysfunction. Cardiac *dSir2* knockdown could promote age-related cardiac contractility debility.

#### Cardiac dSir2 knockdown induced phenotypes similar to heart aging in young Drosophila

**Figure 3:**
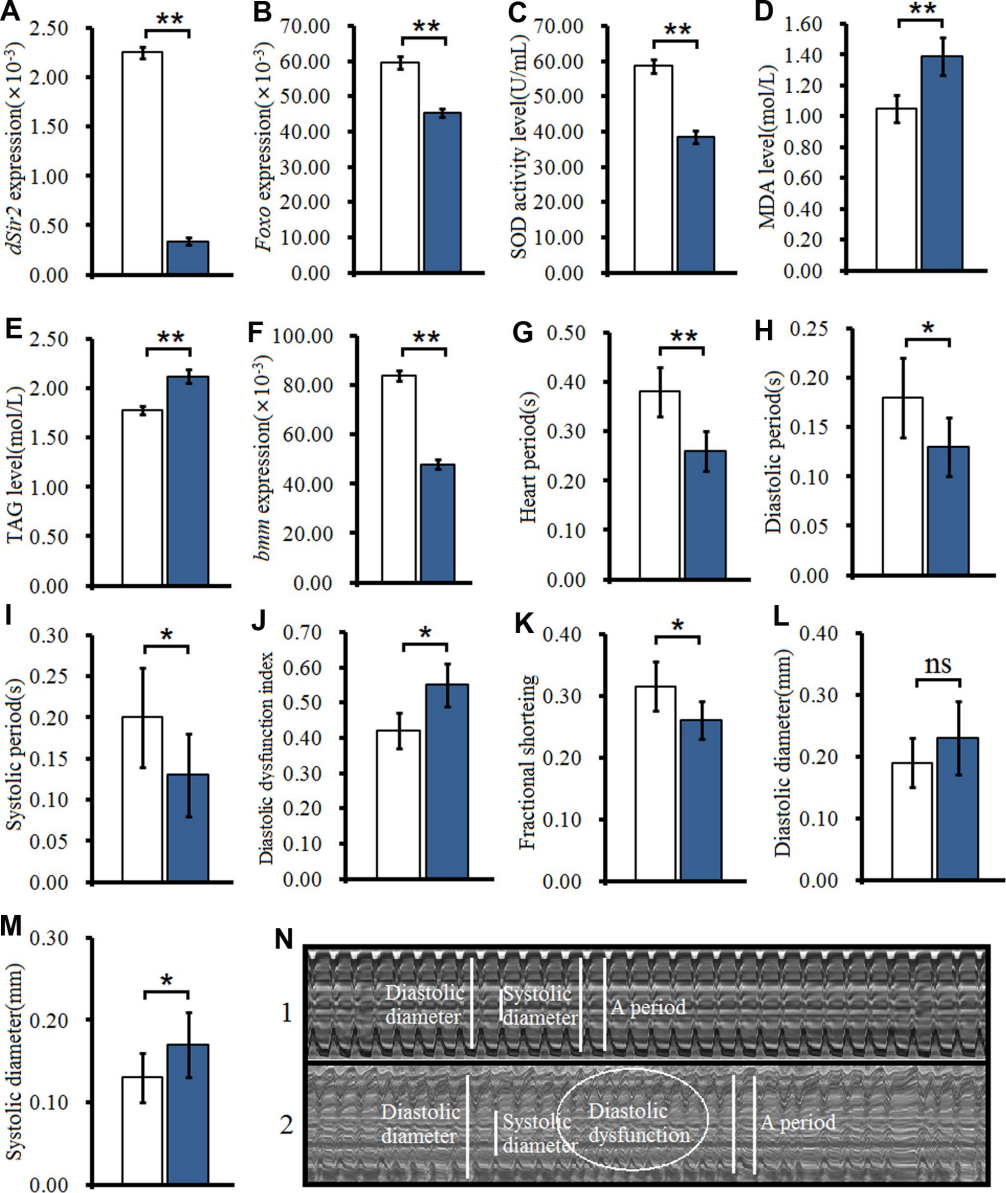
**Effect of cardiac *dSir2* knockdown on young hearts.** (**A**) Cardiac dSir2 expression level. (**B**) Cardiac *Foxo* expression. (**C**) Cardiac SOD activity level. (**D**) Cardiac MDA level. (**E**) Cardiac TAG level. (**F**) Cardiac *bmm* expression. (**G**) Heart period. (**H**) Heart systolic period. (**I**) Heart diastolic period. (**J**) Heart diastolic dysfunction index. The diastolic dysfunction index is diastolic interval standard deviation/diastolic interval median). (**K**) Fractional shortening. (**L**) Diastolic diameter. (**M**) Systolic diameter. (**N**) Microscopic image of cardiac function from M-mode trace. 1: *hand-Gal4>w^11118^* flies; 2: *hand-Gal4>UAS-dSir2^RNAi^* flies; 3: 7-week-old *hand-Gal4>w^11118^* flies; 4: 7-week-old *hand-Gal4>UAS-dSir2-RNAi* flies. It can be observed from 1 and 2 that the cardiac *dSir2* knockdown could reduce heart period and fractional shortening, and increase diastolic dysfunction. Independent-sample *t* tests were used to assess differences between the *hand-Gal4>w^11118^* and *hand-Gal4>UAS-dSir2^RNAi^* flies to explore the effects of cardiac *dSir2* knockdown on the heart. Data are represented as means ± SEM. *P<0.05; **P <0.01. Sample size was the same as in our previous experiments.

To further confirm whether cardiac *dSir2* RNAi could induce age-related changes in the heart, the other cardiac *dSir2* RNAi line was built by *P{KK109370}VIE-260B* and *hand-Gla4* in 1-week-old flies. The results showed that the cardiac *dSir2* expression of *hand-Gal4>UAS-dSir2^RNAi^* flies was significantly lower than that of *hand-Gal4>w^1118^* flies (P<0.01) (a difference of about 5.6-fold; Figure 3A). This suggested that cardiac *dSir2* knockdown was also successfully built by the UAS/hand-Gal4 system. Moreover, the results showed that cardiac *dSir2* knockdown significantly reduced the SOD activity level and *Foxo* expression (P<0.01; Figure 3B and 3C), and notably increased MDA levels in young hearts (P<0.01; Figure 3D). In addition, cardiac *dSir2* knockdown significantly increased cardiac TAG level (+19.1%) and reduced cardiac *bmm* expression (P<0.01; Figure 3E and 3F). Finally, cardiac *dSir2* knockdown significantly reduced heart period, diastolic period, systolic period, and fractional shortening (P<0.05 or P<0.01; Figure 3G–3I, and 3K), and notably increased the diastolic dysfunction index and systolic diameter (P<0.01; Figure 3J, 3M and 3N). These results suggested that cardiac *dSir2* knockdown could increase the incidence of oxidative damage, fat accumulation, and diastolic dysfunction in young hearts, which is similar to the phenotypes associated with aging hearts.

### Cardiac *dSir2* overexpression slows down heart aging in *Drosophila*

**Figure 4:**
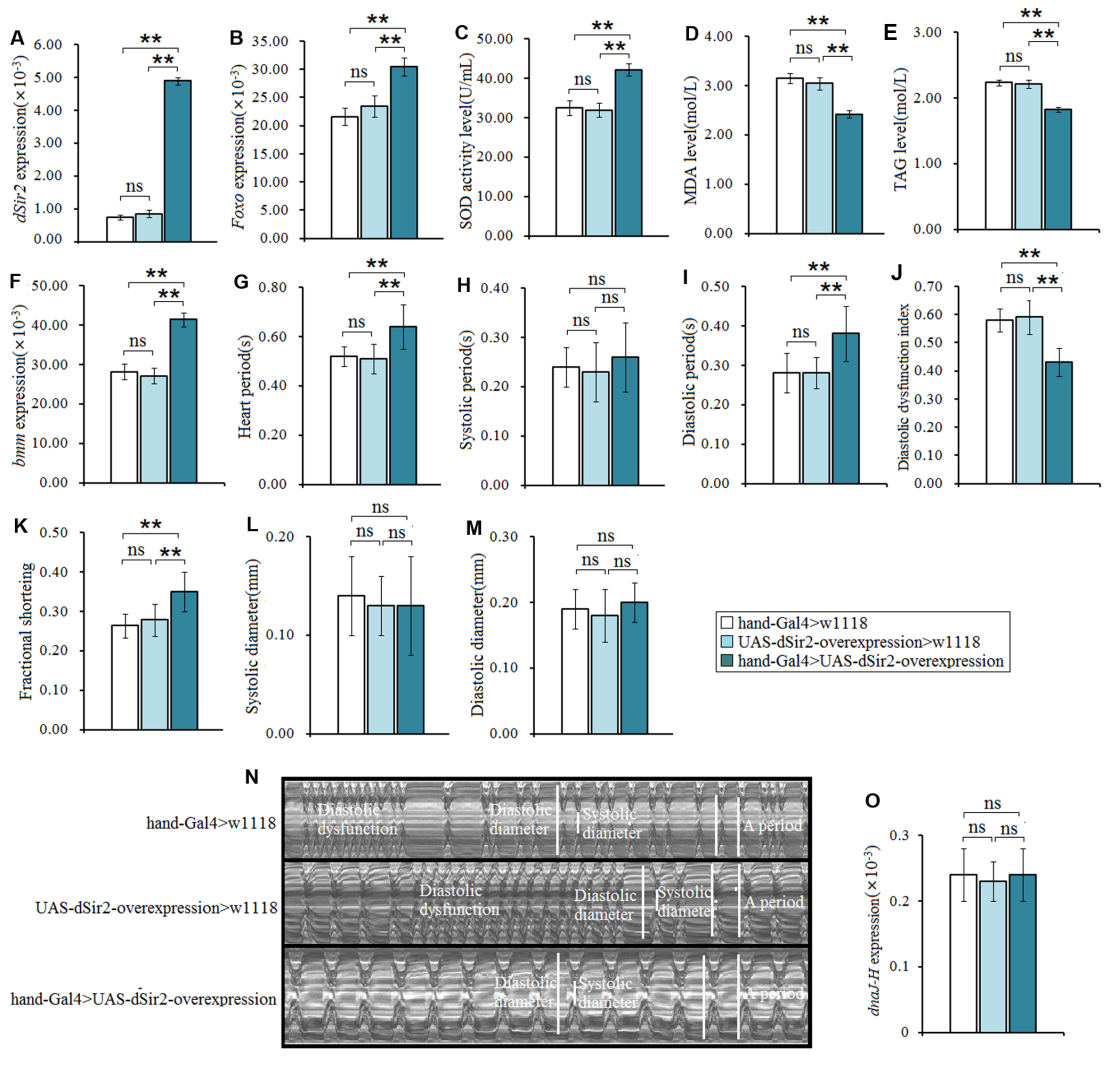
**The influence of cardiac *dSir2* overexpression on the heart in 7-weeek-old flies.** (**A**) Cardiac dSir2 expression. (**B**) Cardiac *Foxo* expression. (**C**) Cardiac SOD activity level. (**D**) Cardiac MDA level. (**E**) Cardiac TAG level. (**F**) Cardiac *bmm* expression. (**G**) Heart period. (**H**) Heart systolic period. (**I**) Heart diastolic period. (**J**) Heart diastolic dysfunction index. The diastolic dysfunction index is diastolic interval standard deviation/diastolic interval median). (**K**) Fractional shortening. (**L**) Diastolic diameter. (**M**) Systolic diameter. (**N**) Microscopic image of cardiac function from M-mode trace. It can be observed that the cardiac *dSir2* overexpression could increase heart period and fractional shortening, and decrease diastolic dysfunction. (**O**) Cardiac *dnaJ-H* expression level. One-way analysis of variance (ANOVA) with least significant difference (LSD) tests were used to identify differences among the *hand-Gal4>w^11118^*, *UAS-dSir2-overexpression>w^11118^*, and *hand-Gal4>UAS-dSir2-overexpression* flies to explore the effects of cardiac *dSir2* overexpression on aging hearts. Data are represented as means ± SEM. *P<0.05; **P <0.01. The sample size was the same as in our previous experiments.

To further confirm whether cardiac *dSir2* could regulate heart aging, the cardiac *dSir2* overexpression line was built by UAS-*dSir2* overexpression (*w^1118^; P{EP}Sirt1^EP2300^ DnaJ-H^EP2300^/CyO*) and *hand-Gla4* in 7-week-old flies. Although our previous results indicated that cardiac *dSir2* knockdown promoted heart aging and aging reduced cardiac *dSir2* expression, it remains unknown whether cardiac *dSir2* overexpression can affect heart aging. The results showed that the cardiac *dSir2* expression of hand-Gal4>UAS-*dSir2*-overexpression flies was significant higher than that of *hand-Gal4>w^1118^* flies (P<0.01) (a difference of about 5.01-fold) (Figure 4A), and the cardiac *dSir2* expression of hand-Gal4>UAS-*dSir2*-overexpression flies was also significantly higher than that of UAS-*dSir2*-overexpression*>w^1118^* flies (P<0.01) (a difference of about 6.6-fold) (Figure 4A). This suggested that the cardiac *dSir2* overexpression was successfully induced by the UAS/hand-Gal4 system in 7-week-old *Drosophila*. Besides, the results showed that cardiac *dSir2* overexpression significantly increased the SOD activity level and *Foxo* expression (P<0.01; Figure 4B and 4C), and notably decreased MDA levels in aging hearts (P<0.01; Figure 4D). Furthermore, cardiac *dSir2* overexpression significantly decreased cardiac TAG level (P<0.01; by 17.6%; Figure 4E), and notably increased cardiac *bmm* expression (P<0.01; Figure 4F). Finally, cardiac *dSir2* overexpression significantly increased heart period, diastolic period, and fractional shortening (P<0.01; Figure 3G, 3I, 3K, and 3N), and notably decreased the diastolic dysfunction index (P<0.01; Figure 3J and 3N). Although a dnaJ-homolog (*dnaJ-H*) gene partially overlaps with *dSir2*, increasing evidence indicates that moderately increased expression of *dSir2* (2.5-fold, 3-fold, and 5-fold) from the native *dSir2* locus can result in lifespan extension without an increase in *dnaJ-H* mRNA expression [52, 53]. The results showed that the heart *dnaJ-H* gene expression was not significantly changed by *dSir2* overexpression (P>0.05; Figure 3O). Therefore, our results suggest that cardiac *dSir2* overexpression could decrease the risk of oxidative damage, fat accumulation, and diastolic dysfunction in aging hearts, thus resisting the phenotypes associated with heart aging.

### Physical exercise’s mitigation of heart aging is related to cardiac *dSir2*-related pathways in *Drosophila*

**Figure 5:**
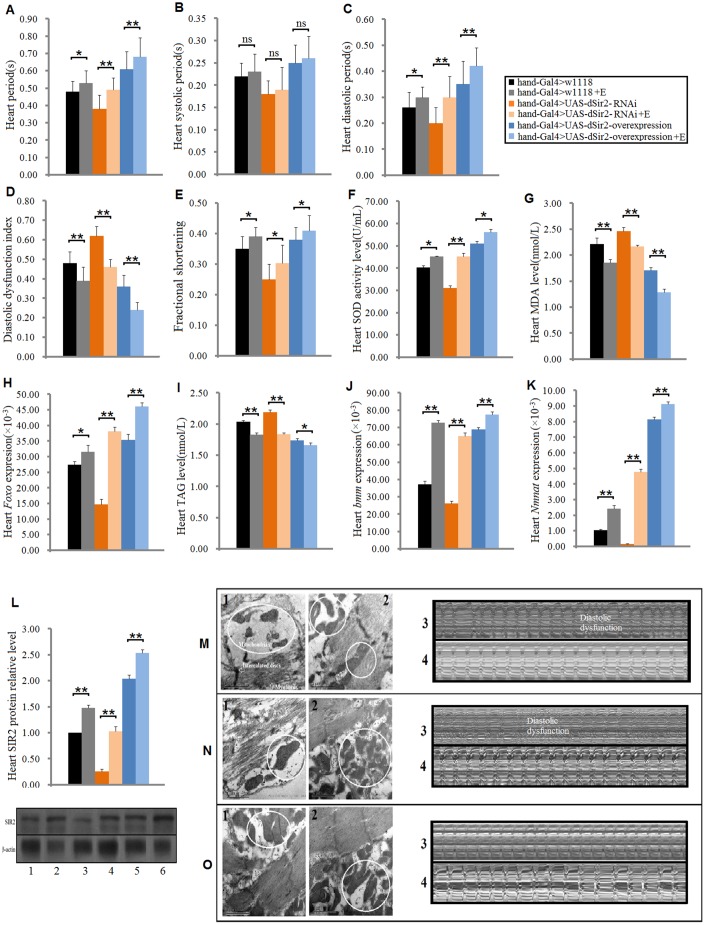
**Effect of exercise training on cardiac functions in cardiac *Sir2* differential-expression and 5-week-old flies.** (**A**) Heart period. (**B**) Systolic period. (**C**) Diastolic period. (**D**) Diastolic dysfunction index. (**E**) Fractional shortening. (**F**) Heart SOD activity level. (**G**) Heart MDA level. (**H**) Heart Foxo expression level. (**I**) Heart TAG level. (**J**) Heart *bmm* expression level. (**K**) Heart *Nmnat* expression level. (**L**) Heart SIR2 protein level. “1” indicates hand-Gal4>w1118, “2” indicates hand-Gal4>w1118+Exercise, “3” indicates hand-Gal4>UAS-dSir2-RNAi, “4” indicates hand-Gal4>UAS-dSir2-RNAi+E, “5” indicates hand-Gal4>UAS-dSir2-overexpression, and “6” indicates hand-Gal4>UAS-dSir2-overexpression+E. Independent-sample *t* tests were used to assess differences between the “Control group” and “Exercise group” in cardiac *dSir2* differential-expression flies to explore the effects of exercise training on the heart. Data are represented as means ± SEM. *P<0.05; **P <0.01. Sample size and repetitions were the same as before. (**M**) Hand-Gal4>w1118 group. (**N**) hand-Gal4>UAS-dSir2-RNAi, and (**O**) hand-Gal4>UAS-dSir2-overexpression. In m, n, and o, 1: ultrastructure image of myocardium in the non-exercise group; 2: ultrastructure image of myocardium in the exercise group; 3: microscopic image of cardiac function in the non-exercise group; and 4: microscopic image of cardiac function in the exercise group. It can be observed from m1, n1, and o1 that the cardiac *dSir2* knockdown could reduce the number of mitochondria and make the arrangement of myofibrils irregular, but cardiac *dSir2* overexpression can increase the number of mitochondria and make the myofibrils more orderly. It can be observed from m3, n3, and o3 that the cardiac *dSir2* knockdown can reduce heart period and increase diastolic dysfunction, and cardiac *dSir2* overexpression can extend heart period and decrease diastolic dysfunction. Moreover, it can be observed from m, n, and o that exercise training can increase the number of heart mitochondria, make the myofibrils more orderly, extend heart period, and reduce diastolic dysfunction in cardiac *dSir2* differential-expression flies.

To study whether the cardiac *dSir2* gene is involved in the delayed age-related decline of heart function after exercise training, cardiac *dSir2* differential-expression flies were trained. In both humans and animals, increasing evidence has showed that exercise training can enhance heart function, which is conducive to delaying the aging of the heart and reducing the occurrence of heart failure [26, 30]. In this study, the results showed that in cardiac *dSir2* differential-expression flies, exercise training significantly prolonged the cardiac period (P<0.05 or P<0.01; Figure 5A); moreover, the systolic period had no significant change after exercise training (P>0.05; Figure 5B) while the diastolic period significantly increased after exercise training (P<0.05 or P<0.01; Figure 4C). Exercise training significantly reduced the diastolic dysfunction index in cardiac *dSir2* differential-expression flies (P<0.01; Figure 4D, 4M–4O). In addition, exercise training significantly increased the cardiac SOD level and *Foxo* expression level (P<0.05 or P<0.01; Figure 4E and 4G) and significantly reduced the cardiac MDA level in cardiac *dSir2* differential-expression flies (P<0.05 or P<0.01; Figure 4F). Furthermore, exercise training significantly decreased the cardiac TAG level, and it up-regulated cardiac *bmm* gene expression in cardiac *dSir2* differential-expression flies (P<0.05 or P<0.01; Figure 4H and 4I). Additionally, exercise training significantly increased the cardiac SIR2 protein level in cardiac *dSir2* differential-expression flies (P<0.01; Figure 4K). Finally, the results showed that cardiac *Nmnat* expression significantly increased after exercise training in cardiac *dSir2* differential-expression flies (P<0.01; Figure 4J). Increasing evidence has shown that exercise trained increases muscle and blood NAD^+^ levels [54, 55]. Cardiac *Nmnat* expression was measured by RT-PCR since NMNAT reversibly catalyzes the important step in the biosynthesis of NAD from ATP and NMN, and overexpression of *Nmnat* has been shown to increase NAD^+^ levels in some tissues [56]. It has been reported that NAD^+^ has an upstream regulation function for dSir2(Smith et al., 2000). Therefore, exercise training may improve dSir2 protein level by increasing NAD^+^ levels in heart *dSir2* differential-expression flies. Therefore, these findings suggested that exercise training could improve cardiac diastolic dysfunction, reduce excessive accumulation of lipids, and decrease oxidative damage. Exercise training combined with cardiac *dSir2* overexpression in old flies could better protect the heart from aging compared to exercise training or cardiac *dSir2* overexpression alone. The mechanism of exercise to delay the age-related decline of cardiac function was closely related to the up-regulation of cardiac *dSir2*, *Foxo*, and *bmm* genes.

### Effect of physical exercise and cardiac *dSir2* differential expression on mobility and lifespan in *Drosophila*

To explore whether the cardiac *Sir2* gene could affect the flies’ climbing ability and longevity, the climbing index and lifespan of the experimental flies were measured. As we all know, the heart is a very important organ to exercise in vertebrate animals since the brain and skeletal muscles get oxygen and nutrients by pumping blood through the heart. Especially during exercise or activity, if the brain and skeletal muscles do not get enough oxygen and nutrients, it will lead to reduced athletic ability and proneness to fatigue [57, 58]. Although the skeletal muscles and brain of flies get their oxygen without having to pump hemolymph through the heart, they need to get nutrition and energy from hemolymph [59, 60]. In this respect, there may be a relationship between heart function and climbing ability in fruit flies. Besides, exercise training has been shown to improve heart function and prevent some heart diseases [25, 36, 61]. Numerous studies have reported that age-related heart disease is the leading cause of death in the elderly [62–64]. These findings suggest that cardiac function is important for mobility and survival in older individuals. However, it remains unclear whether cardiac *dSir2* different expression can affect the fruit fly's motor ability and longevity.

**Figure 6:**
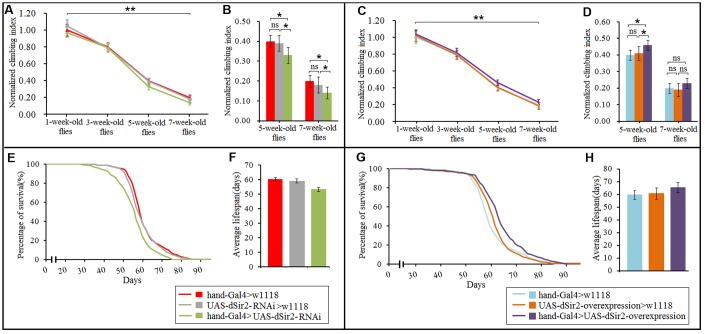
**Effect of cardiac *dSir2* differential-expression on the climbing index and average lifespan in *Drosophila*.** (**A**) The climbing index change curves with aging of cardiac *dSir2* knockdown flies. (**B**) The climbing index of cardiac *dSir2* knockdown flies. The sample size was about 100 flies for each group. (**C**) The climbing index change curves with aging in cardiac *dSir2* overexpression flies. (**D**) The climbing index of cardiac *dSir2* overexpression flies. The sample size was about 100 flies for each group. (**E**) Fly population survival (%) curve of cardiac *dSir2* knockdown flies. The leftmost curve represents the cardiac *dSir2* knockdown group, of which flies had the shortest lifespan. (**F**) The average lifespan of cardiac *dSir2* knockdown flies. The sample size was 200–220 flies for each group. (**G**) Fly population survival (%) curve of cardiac *dSir2* overexpression flies. The leftmost curve represents the cardiac *dSir2* overexpression group, of which flies had the longest lifespan. (**H**) The average lifespan of cardiac *dSir2* overexpression flies. The sample size was 200–220 flies for each group. P-values for lifespan curves were calculated by the log-rank test. Data are represented as means ± SEM. *P<0.05; **P <0.01.

**Figure 7:**
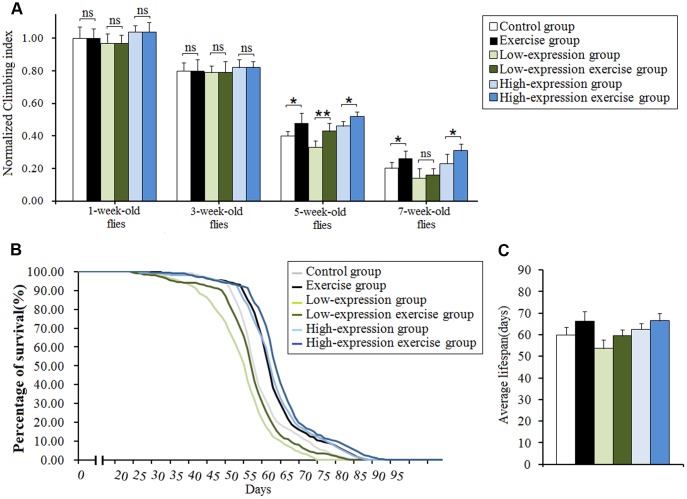
**Effect of exercise and cardiac *Sir2* on the climbing index and average lifespan in *Drosophila*.** (**A**) The climbing index. The sample size was about 100 flies for each group. (**B**) The average lifespan. The sample size was 200–220 flies for each group. (**C**) Percents of survival curve. The rightmost curve represents the cardiac *dSir2* overexpression combined with exercise group, of which flies had the longest lifespan. P-values for lifespan curves were calculated by the log-rank test. Data are represented as means ± SEM. *P<0.05; **P <0.01.

In this study, results showed that aging significantly reduced the climbing index in cardiac *dSir2* differential-expression flies (P<0.01; Figure 6A and 6C). Cardiac *dSir2* knockdown significantly reduced the climbing index in 5-week-old and 7-week-old flies (P<0.05), and also notably decreased the average lifespan of flies (P<0.05; Figure 6B, 6E, and 6F). On the contrary, cardiac *dSir2* overexpression significantly increased the climbing index in 5-week-old flies (P<0.05) and notably increased the average lifespan of flies (P<0.05; Figure 6D, 6G, and 6H). For 1-week-old and 3-week-old flies, cardiac *dSir2* knockdown and overexpression did not remarkably affect the climbing index (P>0.05; Figure 6A and 6C). The results also showed that in 1-week-old and 3-week-old and cardiac *dSir2* differential-expression flies, the climbing index had no significant difference between the exercise group and no exercise group (P>0.05; Figure 7A). This suggested that before exercise training, the climbing index of the exercise group was almost the same as that of the no exercise group. After 2 weeks of exercise training, the climbing index was notably increased in cardiac *dSir2* differential-expression and 5-week-old flies (P<0.05, P<0.01). Exercise training also notably increased the climbing index in cardiac *dSir2* over- or normal-expression and 7-week-old flies (P<0.05), but this effect was not found in cardiac *dSir2* knockdown and 7-week-old flies (P>0.05; Figure 7A). Moreover, exercise remarkably prolonged the average lifespan of cardiac *dSir2* differential-expression flies (P<0.05, P<0.01; Figure 7B and 7C). Therefore, these results suggested that cardiac *Sir2* overexpression and exercise in old flies could improve their mobility and longevity to a certain extent. Cardiac *Sir2* knockdown reduced the mobility and survivability of flies, but these changes could be reversed by exercise training to a certain extent.

## DISCUSSION

In both mammals and fruit flies, the *Sir2/Sirt1* gene is closely related to oxidative stress and lipid metabolism. In mammalian cells, the Sir2 homolog SIRT1 appears to control the cellular response to stress by regulating the FOXO family of Forkhead transcription factors, a family of proteins that function as sensors of the insulin signaling pathway and as regulators of organismal longevity. SIRT1 increased FOXO3's ability to induce cell cycle arrest and resistance to oxidative stress but inhibited FOXO3's ability to induce cell death [65]. In addition, hepatic SIRT1 plays an important role in hepatic fatty acid metabolism, and it has been shown that adenoviral knockdown of SIRT1 reduces expression of fatty acid β-oxidation genes in the liver of fasted mice [66]. SIRT1 can also regulate the brown remodeling of white adipose tissue in response to cold exposure by deacetylation of PPARγ, and SIRT1-dependent deacetylation of PPARγ is required to recruit the brown adipose tissue program coactivator Prdm16 to PPARγ, leading to selective induction of brown adipose tissue genes and repression of white adipose tissue genes [67]. However, it remains unclear whether the cardiac *Sirt1* gene can regulate heart aging.

In *Drosophila*, overexpression of *Sir2* enhances the survival of a number of model organisms undergoing calorie restriction, during which insulin receptor signaling is reduced, a condition that can enhance survivorship during starvation. Besides, defects in peripheral insulin sensitivity, but not in insulin secretion, can account for the reduced insulin signaling in *sir2* mutants, and GAL4-driven expression of *Sir2* in the fat body of wild-type flies is sufficient to reduce TAG levels, which is consistent with previous reports of *SIRT1* overexpression in mice [68]. Moreover, overexpression of *Sir2* in the adult fat body is sufficient to extend the lifespan of male and female Drosophila [53]. Importantly, increased *Sir2* expression and reduced insulin receptor signaling results in an increase in the activity of the transcription factor FOXO [69]. In fly neuronal cells, increased Dyrk1a activates *Sir2* to regulate the deacetylation of FOXO, which potentiates FOXO-induced sNPF/NPY expression and in turn promotes food intake. Recent studies report that modest *Foxo* overexpression is cardioprotective, ameliorating nonpathological functional decline with age in flies [70]. The expression of *Foxo* in myocardial cells autonomously protects the heart from the adverse effects of a high-fat diet [16]. Therefore, *Sir2* is involved in the regulation of oxidative stress and lipid metabolism, possibly through the regulation of *Foxo*. However, it remains unclear whether *Sir2* can upstream regulate *Foxo* in the heart of fruit flies.

In this study, we found that cardiac *Sir2* overexpression increased cardiac *Foxo* expression in aging flies. The activation of FOXO transcription factors leads to the induction of a variety of genes encoding antioxidant proteins, including MnSOD [14]. Therefore, cardiac *Sir2* overexpression increased heart SOD activity level and decreased MDA level via activating the *Sir2/Foxo/*SOD pathway in fly’s hearts; this may improve the antioxidant capacity of myocardial cells and reduce oxidative damage to myocardial cells. On the contrary, cardiac *Sir2* knockdown or aging reduced heart SOD activity and increased heart MDA level via inhibiting the cardiac *Sir2/Foxo/*SOD pathway in *Drosophila*, which may weaken the antioxidant capacity of myocardial cells and increase oxidative damage to myocardial cells. We also found that cardiac *Sir2* overexpression effectively reduced the level of cardiac TAG and increased cardiac *bmm* expression. Because it has been reported that there is no significant increase in heart-specific TAG accumulation in hearts of flies with high-fat diets when overexpressing either *Foxo* or *bmm* [16], cardiac *Sir2* overexpression may decrease cardiac lipid accumulation via activating the cardiac *Sir2/Foxo/bmm* pathway in aging flies. Oppositely, cardiac *Sir2* knockdown or aging caused excessive fat accumulation in the heart by suppressing the cardiac *Sir2/Foxo/bmm* pathway in flies. Furthermore, we found that cardiac *Sir2* overexpression could reduce diastolic dysfunction. Since oxidative stress and excessive lipid accumulation are the main causes of cardiac diastolic dysfunction [71, 72], cardiac *Sir2* overexpression may reduce the risk of diastolic dysfunction by activating the cardiac *Sir2/Foxo/*SOD pathway and *Sir2/Foxo/bmm* pathway. Inversely, cardiac *Sir2* knockdown or aging increased the incidence of diastolic dysfunction by inhibiting the cardiac *Sir2/Foxo/*SOD pathway and *Sir2/Foxo/bmm* pathway in aging flies. Finally, we found that cardiac *Sir2* overexpression could increase heart period and fractional shortening in aging flies, and this indicated that cardiac *Sir2* overexpression enhanced heart contractility. Meanwhile, cardiac *Sir2* knockdown reduced heart period and fractional shortening in aging flies. Therefore, for heart period, cardiac *Sir2* knockdown seemed to resist aging, but this is not actually the case. In order to maintain normal metabolism and cardiac output, the heart may compensate for the reduced fractional shortening by reducing the cardiac period in cardiac *Sir2* knockdown flies. The cardiac fractional shortening may be decreased by lipid accumulation and oxidative damage [71–74]. This suggests that cardiac *Sir2* knockdown attenuated heart contractility. In aging flies, since their metabolic rate drops, the cardiac output may be maintained by decreasing the cardiac fractional shortening or increasing the cardiac period. Of course, aging may also reduce cardiac fractional shortening via increasing lipid accumulation and oxidative damage [3, 5, 6]. Therefore, we cardiac aging is majorly characterized by increased diastolic dysfunction, accumulation of lipid, oxidative stress, and reduced contractility in *Drosophila*. Cardiac *Sir2* overexpression effectively delayed heart aging via activating the *Sir2/Foxo/bmm* pathway and *Sir2/Foxo/*SOD pathway, but cardiac *Sir2* knockdown contributed to the aging process of the heart via inhibiting the *Sir2/Foxo/bmm* pathway and *Sir2/ Foxo/*SOD pathway.

Exercise training can prevent lipid accumulation in both aging mammals and fruit flies. Endogenous TAG represents an important source of fuel for endurance exercise. TAG oxidation increases progressively during exercise, and the specific rate is determined by energy requirements of working muscles, fatty acid delivery to muscle mitochondria, and the oxidation of other substrates. The catecholamine response to exercise increases lipolysis of adipose tissue TAGs, and, presumably, intramuscular TAGs. Moreover, increases in adipose tissue and muscle blood flow decreases fatty acid re-esterification and facilitates the delivery of released fatty acids to skeletal muscle [75]. Therefore, exercise training can accelerate the decomposition of free fatty acids to provide energy. Moreover, it can promote the decomposition of adipose tissue to provide energy, which can effectively prevent excessive accumulation of lipids in tissues and organs such as the liver and heart. In *Drosophila*, it has been reported that endurance exercise can effectively prevent excessive accumulation of lipids in the body and heart induced by a high-fat diet or aging, and this may be related to the benign regulation of *Sir2* expression through exercise [35]. However, it remains unclear whether exercise training resistance to heart lipid accumulation is related to cardiac *Sir2* gene activation. Besides, exercise training can improve cardiac function in both aging mammals and fruit flies. For example, swimming exercise upregulates antioxidant defense capacity and improves structural abnormalities of the senescent female rat heart, and it improves the contractile function of aged myocardium by mitigating the detrimental effects of oxidative stress [61]. Exercise training enhances the SIRT longevity pathway instead of IGF1 survival signaling to improve cardiomyocyte survival [30]. In *Drosophila*, exercise training can reduce age-related decline in mobility and cardiac performance [32]. Fatiguing exercise initiated later in life reduces the incidence of cardiac fibrillation in *Drosophila* [36]. Normal expression or overexpression of CG9940 had a positive influence on the adaptation of cardiac functions, mobility, and lifespan to exercise in aging *Drosophila* [34]. Therefore, since both the cardiac *Sir2/Sirt1* gene and exercise training take part in regulating heart aging, it is important to understand the relationship between them.

In this study, for the first time, we found that in cardiac *dSir2* overexpression, normal expression, knockdown, and 5-week-old flies, exercise training improved cardiac *dSir2* expression and SIR2 protein levels. We also found that in the cardiac *Sir2* differential-expression flies*,* exercise training increased cardiac SOD activity level and decreased MDA levels via activating the cardiac *dSir2/Foxo/*SOD pathway; these changes indicated that exercise training improved the antioxidant capacity of myocardial cells. In addition, exercise decreased cardiac lipid accumulation via activating cardiac the *dSir2/Foxo/bmm* pathway in cardiac *Sir2* differential-expression and 5-week-old flies. Moreover, the risk of diastolic dysfunction was reduced by exercise training in cardiac *Sir2* differential-expression and 5-week-old flies. Finally, we found that exercise training increased heart period and fractional shortening in cardiac *Sir2* differential-expression and 5-week-old flies, and these changes indicated that exercise training enhanced heart contractility. These results suggested that the activation of the cardiac *dSir2/Foxo/*SOD pathway and cardiac *dSir2/Foxo/bmm* pathway may be two important molecular mechanisms through which exercise fights against heart aging.

Although it has been reported that exercise training can enhance the SIRT longevity pathway instead of IGF1 survival signaling to improve cardiomyocyte survival [30], the mechanism by which *dSir2* expression is up-regulated in exercise training remains unclear. Increasing evidence has shown that exercise training increases muscle and blood NAD^+^ levels [54, 55], and the NAD^+^ has an upstream regulation function for *Sir2/Sirt1* [7]. Therefore, we speculate that the *dSir2* expression up-regulated by exercise training in the heart may be achieved by improving the cardiac NAD^+^ level. To test this hypothesis, we examined the expression of the *Nmnat* gene in the heart. Nicotinamide mononucleotide adenylyltransferease (NMNAT), a rate-limiting enzyme present in all organisms, reversibly catalyzes the important step in the biosynthesis of NAD from ATP and NMN, and overexpression of Nmnat has been shown to increase NAD^+^ levels in some tissues [56]. We found that both *dSir2* overexpression and exercise training could improve *Nmnat* expression in the heart. This may be the result of the positive adaptive changes in the synthesis of related genes caused by the long-term high demand for NAD^+^ in the heart. However, this speculation needs to be confirmed by further experiments.

Age-related decline in heart function and heart disease are important causes of reduced physical activity and death in the elderly [22]. Previous findings showed that the cardiac *dSir2* gene can affect heart aging in flies, but it remains unclear whether the cardiac *dSir2* gene can affect their mobility and lifespan. Our results showed that cardiac *dSir2* overexpression and exercise in old flies could improve their mobility and survivability to a certain extent. However, cardiac *dSir2* knockdown reduced mobility and survivability in old flies. In young flies, cardiac *dSir2* gene had no significant effect on climbing ability, and the reason for this may be that changes in heart function induced by cardiac *Sir2* differential expression were not enough to cause hypoxia in tissues such as the brain [57, 58]. Cardiac *Sir2* knockdown may contribute to age-related heart disease and lead to death in the elderly [62–64]. In addition, we found that exercise training improved the mobility in cardiac *dSir2* differential-expression and old flies. Moreover, exercise training prolonged the average lifespan in cardiac *dSir2* differential-expression flies. These findings suggest that exercise training could reverse the decline of mobility and survivability induced by cardiac *dSir2* knockdown to a certain extent in old flies. This may be related to the reconstruction of the NAD/*dSir2* pathway in the heart by exercise training. Besides, exercise training could improve the functioning of other organs and systems, and could prevent other age-related diseases, such as obesity, diabetes, and Parkinson's disease, which could thus extend lifespan in old flies [76, 77]. Therefore, this may be the reason why cardiac *dSir2* overexpression combined with exercise training had the best benefit to lifespan and mobility.

In conclusion, we claim that cardiac *Sir2* overexpression or knockdown can delay or promote heart aging by reducing or increasing age-related oxidative stress, lipid accumulation, diastolic dysfunction, and contractility debility. Activation of the cardiac *Sir2/Foxo/*SOD pathway and cardiac *Sir2/Foxo/bmm* pathway may be two important molecular mechanisms through which exercise fights against heart aging in *Drosophila*.

## MATERIALS AND METHODS

### Fly stocks, diet, and husbandry

*w^1118^* and *hand-Gal4* flies were gifts from Xiu-shan Wu (Heart Development Center of Hunan Normal University). UAS-*dSir2*-overexpression (*w^1118^; P{EP} Sirt1^EP2300^ DnaJ-H^EP2300^/CyO*) flies were obtained from the Bloomington Stock Center. UAS-*dSir2*-RNAi (*w^1118^; P{GD11580}v23201*and *P{KK109370}VIE-260B*) lines were obtained from the Vienna Drosophila RNAi Center. To induce different expression of the *dSir2* gene in the fly heart, male *hand-Gal4* flies were crossed with female *w^1118^* flies, UAS-*dSir2*-overexpression flies, and UAS-*dSir2*-RNAi flies [14]. All UAS and GAL4 insertions were backcrossed into the *w^1118^* line at least 10 times to avoid excess phenotypes affecting the experimental results. Normal food contained 10% yeast, 10% sucrose, and 2% agar [16]. During the experimental time course, flies were housed in a 25°C incubator with 50% humidity and a 12-h light/dark cycle. Fresh food was provided every other day for the duration of the experiment. All flies were raised to the fourth weekend, and were then trained in their fifth week of life since we found that flies were more sensitive to exercise during this stage of life.

### Exercise training device and protocols

When constructing the exercise device, the flies’ natural negative geotaxis behavior was taken to induce upward walking [78]. All exercise group flies started exercise from when they were 22 days old, and underwent a 2-week-long exercise program. Vials with the diet and housing 25 flies each were loaded horizontally into a steel tube that was rotated about its horizontal axis by an electric motor, with a gear regulating its shaft speed. Thus, with the accompanying rotating steel tube, each vial was rotated along its long axis, which made the flies climb (TreadWheel) [31, 33]. Most flies continued to respond by climbing throughout the exercise period. The few that failed to climb were actively walking at the inner wall of the vial [34, 36]. Flies were exercised in vials with a 2.8 cm inner diameter, rotated at 0.16 rev/s. Flies were exercised for 1.5 hours per day.

### Semi-intact *Drosophila* preparation and image analysis

Flies were anesthetized with FlyNap for 2–3 min. The head, ventral thorax, and ventral abdominal cuticle were removed by special glass needles to expose the heart and abdomen. Dissections were done under oxygenated artificial hemolymph. These semi-intact preparations were allowed to equilibrate with oxygenation for 15–20 min before filming. Image analysis of heart contractions was performed using high-speed videos of the preparations. Videos were taken at 120–130 frames per second using a Hamamatsu (McBain Instruments, Chats worth, CA) EM-CCD digital camera on a Leica (McBain Instruments, Chatsworth, CA) DM LFSA microscope with a 10x immersion lens. To get a random sampling of heart function, a single 30-s recording was made for each fly. All images were acquired and contrast enhanced using Simple PCI imaging software (Compix, Sewickley, PA). The heart physiology of the flies was assessed using a semi-automated optical heartbeat analysis program that quantifies the heart period, systolic period, diastolic period, diastolic dysfunction index (diastolic intervals standard deviation/diastolic intervals median), fractional shortening, diastolic diameter, and systolic diameter [79]. The sample size was 30 flies for every group. Since the arrhythmia index calculates the heart period standard deviation normalized to the median heart period, we calculated the diastolic arrhythmicity index as the heart diastolic period standard deviation normalized to the median heart diastolic period.

### ELISA assay

The SOD activity level, MDA level, and TAG level were measured by ELISA assay (Insect SOD activity, MDA, and TAG ELISA Kits, MLBIO). Fly hearts were homogenized in PBS (pH 7.2–7.4). Samples were rapidly frozen with liquid nitrogen and then maintained at 2°C–8°C after melting. Homogenize the samples with grinders, and centrifugation was conducted for 20 min at 2000–3000 rpm. Then we removed the supernatant. The specific steps are as follows: (1) Add standard: set standard wells, and test sample wells. Add standard 50 μl to the standard well. (2) Add sample: add 40μl of sample dilution to the testing sample well, then add 10 μl of the sample (the sample’s final dilution is 5-fold). Avoid touching the well wall as much as possible, and gently mix. (3) Add enzyme: add 100 μl of HRP-conjugate reagent to each well except for the blank well. Don’t add sample and HRP-Conjugate reagent to blank comparison wells, and other each step operation is same. (4) Incubate: after closing the plate with the closure plate membrane, incubate for 60 min at 37°C. (5) Washing: uncover the closure plate membrane, discard liquid, dry by swing, add washing buffer to every well, still for 30 s and then drain; repeat 5 times, and pat dry. (6) Color: add 50 μl of Chromogen Solution A and Chromogen Solution B to each well, evade the light preservation, and let sit for 15 min at 37°C. (7) Stop the reaction: add 50 μl of Stop Solution to each well to stop the reactions (the blue color will change to yellow). (8) Assay: take the blank well as zero, and read absorbance at 450 nm within 15 min of adding Stop Solution.

### qRT-PCR

About 80 hearts from each group were homogenized in Trizol. First, 10 μg of the total RNA was purified by organic solvent extraction from the Trizol (TRIzol, Invitrogen). The purified RNA was treated with DNase I (RNase-free, Roche) and used to produce oligo dT-primed cDNAs (SuperScript II RT, Invitrogen), which were then used as templates for quantitative real-time PCR. The rp49 gene was used as an internal reference for normalizing the quantity of total RNAs. Real-time PCR was performed with SYBR green using an ABI7300 Real-time PCR Instrument (Applied Biosystems). Expression of the various genes was determined by the comparative CT method (ABI Prism 7700 Sequence Detection System User Bulletin #2, Applied Biosystems). Primer sequences of *Sir2* were as follows: F: 5′-GCAGT GCCAGCCC AATAA-3′; R: 5′-AGCCGATCACGATC AGTAGA-3′. Primer sequences of *bmm* were as follows: F: F: 5′-ACTGCAC ATTTCGCTTACCC-3′; R: 5′-GAG AATCCGGGTATGAAGCA-3′. Primer sequences of *Foxo* were as follows: F: 5′-AACAACAGCAGCATC AGCAG-3′; R: 5′-CTGAACCCGAGCATTCAGAT-3′. Primer sequences of *dnaJ-H* were as follows: F: 5′-GCAAGATGGCACACGTAGCTG-3′; R: 5′-CCACTG TAGCAACACGTAATCACC-3′. Primer sequences of *Rp49* were as follows: F: 5 -CTAAGCTG TCGCACAA ATGG-3′; R: 5′-AACTTCTTGAATCCGGTG GG-3′.

### Western blots

Samples of 80 hearts were collected under the indicated conditions at 35 days of age, and homogenized in 100 μL of RIPA buffer containing 1X protease inhibitors (Roche cOmplete Mini EDTA-free protease inhibitor tablets). For dSir2 Western blots, the buffer also contained Calyculin A and okadaic acid. Equivalent amounts of protein were resolved by SDS-PAGE (10% acrylamide), transferred to PVDF membrane overnight at 4°C, and blocked with 5% BSA prior to immunoblotting. Western blots were probed with antibodies for dSir2 (1:50, Developmental Studies Hybridoma Bank #p4A10) and β-actin (1:1000, Cell Signaling #9441). The data shown in the figure5-k are representative of at least three biological replicates. Quantification was performed by measuring protein levels using Image J software. The values reported represent the experimental condition normalized to the control, unless otherwise specified.

### Negative geotaxis assay

The climbing apparatus consisted of an 18-cm-long vial with an inner diameter of 2.8 cm, and flies were allowed to adapt to the vial for 10 min before assessing negative geotaxis. Sponges were placed in the ends of the tube to prevent escape while allowing air exchange. With a light box behind the vials, the rack was tapped down five times and on the fifth, a timed digital camera snapped a picture after 8 s. The extent of climbing could be analyzed visually or by imaging software. Five pictures of each group were taken and averaged to arrive at a fixed score for each vial. The total score for all the flies in a vial was tallied, and then divided by the number of flies in the vial to generate the “climbing index” for that trial. Each vial was subjected to 5 trials, and then the indices from the five trials were averaged [78].

### Lifespan assays

Dead flies were recorded daily. Lifespan was estimated for each fly as the number of days alive from the day of hatching to the day of death. Mean and median lifespan and survival curves were used to characterize the lifespan. Sample sizes were 200 to 210 flies per group [80].

### Statistical analyses

The 1-way analysis of variance (ANOVA) with least significant difference (LSD) tests was used to identify differences among the *hand-Gal4>w^11118^*, *UAS-dSir2-overexpression>w^11118^*, and *hand-Gal4>UAS-dSir2-overexpression* flies. Independent-sample *t* tests were used to assess differences between the *hand-Gal4>w^11118^* and *hand-Gal4>UAS-dSir2-RNAi* flies. Analyses were performed using the Statistical Package for the Social Sciences (SPSS) version 16.0 for Windows (SPSS Inc., Chicago, USA), with statistical significance set at *P<0.05*. Data are represented as means ± SEM.

## References

[1] McCullough PA, Philbin EF, Spertus JA, Kaatz S, Sandberg KR, Weaver WD, and Resource Utilization Among Congestive Heart Failure (REACH) Study. Confirmation of a heart failure epidemic: findings from the Resource Utilization Among Congestive Heart Failure (REACH) study. J Am Coll Cardiol. 2002; 39:60–69. 10.1016/S0735-1097(01)01700-4 11755288

[2] Ouwerkerk W, Voors AA, Zwinderman AH. Factors influencing the predictive power of models for predicting mortality and/or heart failure hospitalization in patients with heart failure. JACC Heart Fail. 2014; 2:429–36. 10.1016/j.jchf.2014.04.006 25194294

[3] Drosatos K. Fatty old hearts: role of cardiac lipotoxicity in age-related cardiomyopathy. Pathobiol Aging Age Relat Dis. 2016; 6:32221. 10.3402/pba.v6.32221 27558317 PMC4996860

[4] Klassen MP, Peters CJ, Zhou S, Williams HH, Jan LY, Jan YN. Age-dependent diastolic heart failure in an in vivo *Drosophila* model. eLife. 2017; 6:e20851. 10.7554/eLife.20851 28328397 PMC5362267

[5] Obas V, Vasan RS. The aging heart. Clin Sci (Lond). 2018; 132:1367–82. 10.1042/CS20171156 29986877

[6] Pan B, Xu ZW, Xu Y, Liu LJ, Zhu J, Wang X, Nan C, Zhang Z, Shen W, Huang XP, Tian J. Diastolic dysfunction and cardiac troponin I decrease in aging hearts. Arch Biochem Biophys. 2016; 603:20–28. 10.1016/j.abb.2016.05.008 27184165 PMC5448708

[7] Smith JS, Brachmann CB, Celic I, Kenna MA, Muhammad S, Starai VJ, Avalos JL, Escalante-Semerena JC, Grubmeyer C, Wolberger C, Boeke JD. A phylogenetically conserved NAD+-dependent protein deacetylase activity in the Sir2 protein family. Proc Natl Acad Sci USA. 2000; 97:6658–63. 10.1073/pnas.97.12.6658 10841563 PMC18692

[8] Frankel S, Rogina B. Sir2, caloric restriction and aging. Pathol Biol (Paris). 2006; 54:55–57. 10.1016/j.patbio.2005.04.003 15935564

[9] Shen T, Ding L, Ruan Y, Qin W, Lin Y, Xi C, Lu Y, Dou L, Zhu Y, Cao Y, Man Y, Bian Y, Wang S, et al. SIRT1 functions as an important regulator of estrogen-mediated cardiomyocyte protection in angiotensin II-induced heart hypertrophy. Oxid Med Cell Longev. 2014; 2014:713894. 10.1155/2014/713894 25614777 PMC4295138

[10] Passariello CL, Zini M, Nassi PA, Pignatti C, Stefanelli C. Upregulation of SIRT1 deacetylase in phenylephrine-treated cardiomyoblasts. Biochem Biophys Res Commun. 2011; 407:512–16. 10.1016/j.bbrc.2011.03.049 21414296

[11] Gu XS, Wang ZB, Ye Z, Lei JP, Li L, Su DF, Zheng X. Resveratrol, an activator of SIRT1, upregulates AMPK and improves cardiac function in heart failure. Genet Mol Res. 2014; 13:323–35. 10.4238/2014.January.17.17 24535859

[12] Lu TM, Tsai JY, Chen YC, Huang CY, Hsu HL, Weng CF, Shih CC, Hsu CP. Downregulation of Sirt1 as aging change in advanced heart failure. J Biomed Sci. 2014; 21:57. 10.1186/1423-0127-21-57 24913149 PMC4113120

[13] Alcendor RR, Gao S, Zhai P, Zablocki D, Holle E, Yu X, Tian B, Wagner T, Vatner SF, Sadoshima J. Sirt1 regulates aging and resistance to oxidative stress in the heart. Circ Res. 2007; 100:1512–21. 10.1161/01.RES.0000267723.65696.4a 17446436

[14] Koh H, Kim H, Kim MJ, Park J, Lee HJ, Chung J. Silent information regulator 2 (Sir2) and Forkhead box O (FOXO) complement mitochondrial dysfunction and dopaminergic neuron loss in Drosophila PTEN-induced kinase 1 (PINK1) null mutant. J Biol Chem. 2012; 287:12750–58. 10.1074/jbc.M111.337907 22378780 PMC3339960

[15] Ma J, Chen L, Song D, Zhang Y, Chen T, Niu P. SIRT1 attenuated oxidative stress induced by methyl *tert*-butyl ether in HT22 cells. Toxicol Res (Camb). 2017; 6:290–96. 10.1039/C7TX00016B 30090498 PMC6062265

[16] Birse RT, Choi J, Reardon K, Rodriguez J, Graham S, Diop S, Ocorr K, Bodmer R, Oldham S. High-fat-diet-induced obesity and heart dysfunction are regulated by the TOR pathway in Drosophila. Cell Metab. 2010; 12:533–44. 10.1016/j.cmet.2010.09.014 21035763 PMC3026640

[17] Blice-Baum A, Kaushik G, Viswanathan M, Zambon A, Engler A, Bodmer R, Cammarato A. Overexpression of Foxo in the Heart Ameliorates Performance Decline through Enhanced UPS Processing in Aging Drosophila. Biophys J. 2015 (Suppl 1); 108:361a 10.1016/j.bpj.2014.11.1979

[18] Diop SB, Bodmer R. Drosophila as a model to study the genetic mechanisms of obesity-associated heart dysfunction. J Cell Mol Med. 2012; 16:966–71. 10.1111/j.1582-4934.2012.01522.x 22303936 PMC3454526

[19] Hori YS, Kuno A, Hosoda R, Horio Y. Regulation of FOXOs and p53 by SIRT1 modulators under oxidative stress. PLoS One. 2013; 8:e73875. 10.1371/journal.pone.0073875 24040102 PMC3770600

[20] Lin CH, Lin CC, Ting WJ, Pai PY, Kuo CH, Ho TJ, Kuo WW, Chang CH, Huang CY, Lin WT. Resveratrol enhanced FOXO3 phosphorylation via synergetic activation of SIRT1 and PI3K/Akt signaling to improve the effects of exercise in elderly rat hearts. Age (Dordr). 2014; 36:9705. 10.1007/s11357-014-9705-5 25158994 PMC4453936

[21] Zhang T, Berrocal JG, Frizzell KM, Gamble MJ, DuMond ME, Krishnakumar R, Yang T, Sauve AA, Kraus WL. Enzymes in the NAD+ salvage pathway regulate SIRT1 activity at target gene promoters. J Biol Chem. 2009; 284:20408–17. 10.1074/jbc.M109.016469 19478080 PMC2740465

[22] Roh J, Rhee J, Chaudhari V, Rosenzweig A. The Role of Exercise in Cardiac Aging: From Physiology to Molecular Mechanisms. Circ Res. 2016; 118:279–95. 10.1161/CIRCRESAHA.115.305250 26838314 PMC4914047

[23] Murawski U, Kriesten K, Egge H. Age-related changes of lipid fractions and total fatty acids in liver lipids and heart lipids of female and male rats aged 37-1200 days (liver) and 331-1200 days (heart). Comp Biochem Physiol B. 1990; 96:271–89. 10.1016/0305-0491(90)90375-4 2361362

[24] Zhang B. Effects of adopting exercise intervention for adolescents with simple obesity on heart rate, blood pressure and lipid metabolism. Basic Clin Pharmacol Toxicol. 2017; 121:7–7.

[25] Nolte K, Schwarz S, Gelbrich G, Mensching S, Siegmund F, Wachter R, Hasenfuss G, Düngen HD, Herrmann-Lingen C, Halle M, Pieske B, Edelmann F. Effects of long-term endurance and resistance training on diastolic function, exercise capacity, and quality of life in asymptomatic diastolic dysfunction vs. heart failure with preserved ejection fraction. ESC Heart Fail. 2014; 1:59–74. 10.1002/ehf2.12007 28834666

[26] Sandri M, Kozarez I, Adams V, Mangner N, Höllriegel R, Erbs S, Linke A, Möbius-Winkler S, Thiery J, Kratzsch J, Teupser D, Mende M, Hambrecht R, et al. Age-related effects of exercise training on diastolic function in heart failure with reduced ejection fraction: the Leipzig Exercise Intervention in Chronic Heart Failure and Aging (LEICA) Diastolic Dysfunction Study. Eur Heart J. 2012; 33:1758–68. 10.1093/eurheartj/ehr469 22267243

[27] Byrne JA, Grieve DJ, Cave AC, Shah AM. Oxidative stress and heart failure. Arch Mal Coeur Vaiss. 2003; 96:214–21. 12722552

[28] Ghorbanzadeh V, Mohammadi M, Mohaddes G, Dariushnejad H, Chodari L, Mohammadi S. Protective effect of crocin and voluntary exercise against oxidative stress in the heart of high-fat diet-induced type 2 diabetic rats. Physiol Int. 2016; 103:459–68. 10.1556/2060.103.2016.4.6 28229629

[29] Lund J, Hafstad AD, Boardman NT, Rossvoll L, Rolim NP, Ahmed MS, Florholmen G, Attramadal H, Wisløff U, Larsen TS, Aasum E. Exercise training promotes cardioprotection through oxygen-sparing action in high fat-fed mice. Am J Physiol Heart Circ Physiol. 2015; 308:H823–29. 10.1152/ajpheart.00734.2014 25637547

[30] Lai CH, Ho TJ, Kuo WW, Day CH, Pai PY, Chung LC, Liao PH, Lin FH, Wu ET, Huang CY. Exercise training enhanced SIRT1 longevity signaling replaces the IGF1 survival pathway to attenuate aging-induced rat heart apoptosis. Age (Dordr). 2014; 36:9706. 10.1007/s11357-014-9706-4 25148910 PMC4453937

[31] Lowman KE, Wyatt BJ, Cunneely OP, Reed LK. The TreadWheel: Interval Training Protocol for Gently Induced Exercise in Drosophila melanogaster. J Vis Exp. 2018; 136:57788. 10.3791/57788 29939171 PMC6101642

[32] Piazza N, Gosangi B, Devilla S, Arking R, Wessells R. Exercise-training in young Drosophila melanogaster reduces age-related decline in mobility and cardiac performance. PLoS One. 2009; 4:e5886. 10.1371/journal.pone.0005886 19517023 PMC2691613

[33] Sujkowski A, Wessells R. Using Drosophila to Understand Biochemical and Behavioral Responses to Exercise. Exerc Sport Sci Rev. 2018; 46:112–20. 10.1249/JES.0000000000000139 29346165 PMC5856617

[34] Wen DT, Zheng L, Ni L, Wang H, Feng Y, Zhang M. The expression of CG9940 affects the adaptation of cardiac function, mobility, and lifespan to exercise in aging Drosophila. Exp Gerontol. 2016; 83:6–14. 10.1016/j.exger.2016.07.006 27448710

[35] Wen DT, Zheng L, Yang F, Li HZ, Hou WQ. Endurance exercise prevents high-fat-diet induced heart and mobility premature aging and *dsir*2 expression decline in aging *Drosophila* Oncotarget. 2017; 9:7298–311. 10.18632/oncotarget.23292 29484111 PMC5800903

[36] Zheng L, Feng Y, Wen DT, Wang H, Wu XS. Fatiguing exercise initiated later in life reduces incidence of fibrillation and improves sleep quality in Drosophila. Age (Dordr). 2015; 37:9816. 10.1007/s11357-015-9816-7 26206392 PMC4512962

[37] Busson D, Pret AM. GAL4/UAS targeted gene expression for studying Drosophila Hedgehog signaling. Methods Mol Biol. 2007; 397:161–201. 10.1007/978-1-59745-516-9_13 18025721

[38] Ou H, Lei T. A novel strategy for conditional gene knockout based on ΦC31 integrase and Gal4/UAS system in Drosophila. IUBMB Life. 2013; 65:144–48. 10.1002/iub.1119 23297111

[39] Yin Y, Han W, Cao Y. Association between activities of SOD, MDA and Na^+^-K^+^-ATPase in peripheral blood of patients with acute myocardial infarction and the complication of varying degrees of arrhythmia. Hellenic J Cardiol. 2018. [Epub ahead of print]. 10.1016/j.hjc.2018.04.003 29702256

[40] Jing L, Wang Y, Zhao XM, Zhao B, Han JJ, Qin SC, Sun XJ. Cardioprotective Effect of Hydrogen-rich Saline on Isoproterenol-induced Myocardial Infarction in Rats. Heart Lung Circ. 2015; 24:602–10. 10.1016/j.hlc.2014.11.018 25533677

[41] Zhao YB, Wang YZ, Yue YH, Zhao WC, Feng GX. Variation of plasma levels of endothelin, calcitonin gene-related peptide, nitric oxide, and malondialdehyde in acute myocardial ischemia reperfusion injury in a rabbit model. Genet Mol Res. 2015; 14:5577–84. 10.4238/2015.May.25.9 26125755

[42] Cao P, Shen D, Zhong Y, Yuan GX. Effect of Catalpol on MDA, SOD and GSH-Px of Vascular Endothelial Cell in Aging Rats Induced by D-galactose. J Am Geriatr Soc. 2018; 66:S490–490.

[43] Brown KA, Didion SP, Andresen JJ, Faraci FM. Mn-SOD deficient mice exhibit increased oxidative stress and vascular dysfunction with aging. FASEB J. 2005; 19:A201–201.

[44] Duryee MJ, Klassen LW, Schaffert CS, Tuma DJ, Hunter CD, Garvin RP, Anderson DR, Thiele GM. Malondialdehyde-acetaldehyde adduct is the dominant epitope after MDA modification of proteins in atherosclerosis. Free Radic Biol Med. 2010; 49:1480–86. 10.1016/j.freeradbiomed.2010.08.001 20696236 PMC2952714

[45] Chen H, Xu Y, Wang J, Zhao W, Ruan H. Baicalin ameliorates isoproterenol-induced acute myocardial infarction through iNOS, inflammation and oxidative stress in rat. Int J Clin Exp Pathol. 2015; 8:10139–47. 26617721 PMC4637536

[46] Cui X, Gong J, Han H, He L, Teng Y, Tetley T, Sinharay R, Chung KF, Islam T, Gilliland F, Grady S, Garshick E, Li Z, Zhang JJ. Relationship between free and total malondialdehyde, a well-established marker of oxidative stress, in various types of human biospecimens. J Thorac Dis. 2018; 10:3088–3097. 10.21037/jtd.2018.05.92 29997978 PMC6006110

[47] Liu N, Chen J, Gao D, Li W, Zheng D. Astaxanthin attenuates contrast agent-induced acute kidney injury in vitro and in vivo via the regulation of SIRT1/FOXO3a expression. Int Urol Nephrol. 2018; 50:1171–80. 10.1007/s11255-018-1788-y 29368247

[48] Klotz LO. Redox regulation of FOXO transcription factors - role of glutathione. Free Radic Biol Med. 2017; 108:S5–5. 10.1016/j.freeradbiomed.2017.04.045.

[49] Pamplona R, Portero-Otín M, Riba D, Ledo F, Gredilla R, Herrero A, Barja G. Heart fatty acid unsaturation and lipid peroxidation, and aging rate, are lower in the canary and the parakeet than in the mouse. Aging (Milano). 1999; 11:44–49. 10.1007/BF03399636 10337442

[50] Men TT, Thanh DN, Yamaguchi M, Suzuki T, Hattori G, Arii M, Huy NT, Kamei K. A Drosophila Model for Screening Antiobesity Agents. Biomed Res Int. 2016; 2016:6293163. 10.1155/2016/6293163 27247940 PMC4876200

[51] Czuriga D, Paulus WJ, Czuriga I, Édes I, Papp Z, Borbély A. Cellular mechanisms for diastolic dysfunction in the human heart. Curr Pharm Biotechnol. 2012; 13:2532–38. 10.2174/1389201011208062532 22280428

[52] Burnett C, Valentini S, Cabreiro F, Goss M, Somogyvári M, Piper MD, Hoddinott M, Sutphin GL, Leko V, McElwee JJ, Vazquez-Manrique RP, Orfila AM, Ackerman D, et al. Absence of effects of Sir2 overexpression on lifespan in C. elegans and Drosophila. Nature. 2011; 477:482–85. 10.1038/nature10296 21938067 PMC3188402

[53] Hoffmann J, Romey R, Fink C, Yong L, Roeder T. Overexpression of Sir2 in the adult fat body is sufficient to extend lifespan of male and female Drosophila. Aging (Albany NY). 2013; 5:315–27. 10.18632/aging.100553 23765091 PMC3651523

[54] Fukuwatari T, Shibata K, Ishihara K, Fushiki T, Sugimoto E. Elevation of blood NAD level after moderate exercise in young women and mice. J Nutr Sci Vitaminol (Tokyo). 2001; 47:177–79. 10.3177/jnsv.47.177 11508711

[55] White AT, Schenk S. NAD(+)/NADH and skeletal muscle mitochondrial adaptations to exercise. Am J Physiol Endocrinol Metab. 2012; 303:E308–21. 10.1152/ajpendo.00054.2012 22436696 PMC3423123

[56] Jayaram HN, Kusumanchi P, Yalowitz JA. NMNAT expression and its relation to NAD metabolism. Curr Med Chem. 2011; 18:1962–72. 10.2174/092986711795590138 21517776

[57] Naumenko SE, Belavin AS, Kim SF. [Blood oxygen transport function during ftorotan application in patients with ischemic heart disease and low cardiac output]. Anesteziol Reanimatol. 2003; 18:50–53. 12918203

[58] Shlyk SV, Terent’ev VP, Mikashinovich ZI. [The oxygen-transport function of the blood and cell metabolism in patients with heart failure of different origins]. Ter Arkh. 1999; 71:78–80. 10222563

[59] Lee G, Park JH. Hemolymph sugar homeostasis and starvation-induced hyperactivity affected by genetic manipulations of the adipokinetic hormone-encoding gene in Drosophila melanogaster. Genetics. 2004; 167:311–23. 10.1534/genetics.167.1.311 15166157 PMC1470856

[60] Terashima J, Takaki K, Sakurai S, Bownes M. Nutritional status affects 20-hydroxyecdysone concentration and progression of oogenesis in Drosophila melanogaster. J Endocrinol. 2005; 187:69–79. 10.1677/joe.1.06220 16214942

[61] Ozturk N, Olgar Y, Er H, Kucuk M, Ozdemir S. Swimming exercise reverses aging-related contractile abnormalities of female heart by improving structural alterations. Cardiol J. 2017; 24:85–93. 10.5603/CJ.a2016.0069 27665854

[62] Agarwal R, Norton JM, Konty K, Zimmerman R, Glover M, Lekiachvili A, McGruder H, Malarcher A, Casper M, Mensah GA, Thorpe L. Overreporting of deaths from coronary heart disease in New York City hospitals, 2003. Prev Chronic Dis. 2010; 7:A47. 20394686 PMC2879979

[63] Arsenos P, Gatzoulis K, Manis G, Gialernios T, Dilaveris P, Tsiachris D, Archontakis S, Kartsagoulis E, Mytas D, Stefanadis C. Decreased scale-specific heart rate variability after multiresolution wavelet analysis predicts sudden cardiac death in heart failure patients. Int J Cardiol. 2012; 154:358–60. 10.1016/j.ijcard.2011.11.007 22133464

[64] Gonzalez-Loyola F, Abellana R, Verdú-Rotellar JM, Bustamante Rangel A, Clua-Espuny JL, Muñoz MA. Mortality in heart failure with atrial fibrillation: role of digoxin and diuretics. Eur J Clin Invest. 2018; 48:e13014. 10.1111/eci.13014 30091171

[65] Brunet A, Sweeney LB, Sturgill JF, Chua KF, Greer PL, Lin Y, Tran H, Ross SE, Mostoslavsky R, Cohen HY, Hu LS, Cheng HL, Jedrychowski MP, et al. Stress-dependent regulation of FOXO transcription factors by the SIRT1 deacetylase. Science. 2004; 303:2011–15. 10.1126/science.1094637 14976264

[66] Ye X, Li M, Hou T, Gao T, Zhu WG, Yang Y. Sirtuins in glucose and lipid metabolism. Oncotarget. 2017; 8:1845–59. 10.18632/oncotarget.12157 27659520 PMC5352102

[67] Qiang L, Wang L, Kon N, Zhao W, Lee S, Zhang Y, Rosenbaum M, Zhao Y, Gu W, Farmer SR, Accili D. Brown remodeling of white adipose tissue by SirT1-dependent deacetylation of Pparγ. Cell. 2012; 150:620–32. 10.1016/j.cell.2012.06.027 22863012 PMC3413172

[68] Palu RA, Thummel CS. Sir2 Acts through Hepatocyte Nuclear Factor 4 to maintain insulin Signaling and Metabolic Homeostasis in Drosophila. PLoS Genet. 2016; 12:e1005978. 10.1371/journal.pgen.1005978 27058248 PMC4825955

[69] Slade JD, Staveley BE. Extended longevity and survivorship during amino-acid starvation in a Drosophila Sir2 mutant heterozygote. Genome. 2016; 59:311–18. 10.1139/gen-2015-0213 27074822

[70] Blice-Baum AC, Zambon AC, Kaushik G, Viswanathan MC, Engler AJ, Bodmer R, Cammarato A. Modest overexpression of FOXO maintains cardiac proteostasis and ameliorates age-associated functional decline. Aging Cell. 2017; 16:93–103. 10.1111/acel.12543 28090761 PMC5242305

[71] Abdurrachim D, Ciapaite J, Wessels B, Nabben M, Luiken JJ, Nicolay K, Prompers JJ. Cardiac diastolic dysfunction in high-fat diet fed mice is associated with lipotoxicity without impairment of cardiac energetics in vivo. Biochim Biophys Acta. 2014; 1842:1525–37. 10.1016/j.bbalip.2014.07.016 25086219

[72] Choi YS, de Mattos AB, Shao D, Li T, Nabben M, Kim M, Wang W, Tian R, Kolwicz SC Jr. Preservation of myocardial fatty acid oxidation prevents diastolic dysfunction in mice subjected to angiotensin II infusion. J Mol Cell Cardiol. 2016; 100:64–71. 10.1016/j.yjmcc.2016.09.001 27693463 PMC5154855

[73] Li L, Zhao L, Yi-Ming W, Yu YS, Xia CY, Duan JL, Su DF. Sirt1 hyperexpression in SHR heart related to left ventricular hypertrophy. Can J Physiol Pharmacol. 2009; 87:56–62. 10.1139/Y08-099 19142216

[74] Pillai JB, Gupta M, Rajamohan SB, Lang R, Raman J, Gupta MP. Poly(ADP-ribose) polymerase-1-deficient mice are protected from angiotensin II-induced cardiac hypertrophy. Am J Physiol Heart Circ Physiol. 2006; 291:H1545–53. 10.1152/ajpheart.01124.2005 16632544

[75] Horowitz JF, Klein S. Lipid metabolism during endurance exercise. Am J Clin Nutr. 2000 (2 Suppl); 72:558S–63S. 10.1093/ajcn/72.2.558S 10919960

[76] Intlekofer KA, Cotman CW. Exercise counteracts declining hippocampal function in aging and Alzheimer’s disease. Neurobiol Dis. 2013; 57:47–55. 10.1016/j.nbd.2012.06.011 22750524

[77] Touati S, Meziri F, He Y, Montezano A, Rhian RT, Pascal L. Exercise reverses endothelial dysfunction, oxidative stress and inflammation in rats with high-fat diet-induced obesity. Fundam Clin Pharmacol. 2010; 24:37–37.20002751

[78] Tinkerhess MJ, Ginzberg S, Piazza N, Wessells RJ. Endurance Training Protocol and Longitudinal Performance Assays for Drosophila melanogaster. J Vis Exp. 2012; 61:3786. 10.3791/3786 22472601 PMC3460591

[79] Fink M, Callol-Massot C, Chu A, Ruiz-Lozano P, Izpisua Belmonte JC, Giles W, Bodmer R, Ocorr K. A new method for detection and quantification of heartbeat parameters in Drosophila, zebrafish, and embryonic mouse hearts. Biotechniques. 2009; 46:101–13. 10.2144/000113078 19317655 PMC2855226

[80] He Y, Jasper H. Studying aging in Drosophila. Methods. 2014; 68:129–33. 10.1016/j.ymeth.2014.04.008 24751824 PMC4066732

